# Assessing the role of cervical cancer awareness in shaping attitudes toward the disease among Palestinian women

**DOI:** 10.1038/s41598-025-08068-1

**Published:** 2025-07-01

**Authors:** Mohamedraed Elshami, Lana Khatib, Ibrahim Al-Slaibi, Mohammed Alser, Hanan Abukmail, Afnan Radaydeh, Alaa Alfuqaha, Mariam Thalji, Salma Khader, Manar Zamel, Nour Fannoun, Bisan Ahmad, Lina Kassab, Hiba Khrishi, Deniz Houssaini, Nour Abed, Aya Nammari, Tumodir Abdallah, Zaina Alqudwa, Shahd Idais, Ghaid Tanbouz, Ma’alem Hajajreh, Hala Abu Selmiyh, Zakia Abo-Hajouj, Haya Hebi, Refqa Najeeb Skaik, Lama Hammoud, Saba Rjoub, Hadeel Ayesh, Toqa Rjoub, Rawan Zakout, Amany Alser, Shurouq I. Albarqi, Mysoon Abu-El-Noor, Nasser Abu-El-Noor, Bettina Bottcher

**Affiliations:** 1 Division of Surgical Oncology, Department of Surgery, University Hospitals Cleveland Medical Center, 11100 Euclid Avenue, Lakeside 7100, Cleveland, OH 44106 USA; 2 Ministry of Health, Gaza, Palestine; 3 Ministry of Health, Nablus, Palestine; 4 Almakassed Hospital, Jerusalem, Palestine; 5 The United Nations Relief and Works Agency for Palestine Refugees in the Near East, Gaza, Palestine; 6 Department of Public Health and Primary Care, University of Cambridge, Cambridge, UK; 7 Harvard Medical School, Boston, MA USA; 8 International Medical Corps, Gaza, Palestine; 9 Ministry of Health, Bethlehem, Palestine; 10 Faculty of Graduate Studies, An-Najah National University, Nablus, Palestine; 11 Faculty of Medicine, Al-Quds University, Jerusalem, Palestine; 12 Hebron Governmental Hospital, Hebron, Palestine; 13 Faculty of Medicine, An-Najah National University, Nablus, Palestine; 14 Faculty of Pharmacy, Alazhar University of Gaza, Gaza, Palestine; 15 Faculty of Medicine, Islamic University of Gaza, Gaza, Palestine; 16 Faculty of Dentistry and Dental Surgery, Al-Quds University, Jerusalem, Palestine; 17 Faculty of Medicine, Alazhar University of Gaza, Gaza, Palestine; 18 Alia Hospital, Hebron, Palestine; 19 Al-Shiffa Hospital, Gaza, Palestine; 20 Faculty of Nursing, Islamic University of Gaza, Gaza, Palestine

**Keywords:** Cervical cancer, Symptoms, Risk factors, Causation myths, Health behaviors, Palestine, Health care, Cancer

## Abstract

Attitudes toward a disease may influence the perception and interpretation of symptoms and treatment options. This study aimed to assess the role of cervical cancer (CC) awareness in shaping attitudes toward the disease among Palestinian women. This cross-sectional study was conducted from July 2019 to March 2020, and women were recruited from governmental hospitals, primary healthcare centers, and public places utilizing convenience sampling. Modified, translated-into-Arabic versions of the CC Awareness Measure and Cancer Awareness Measure-Mythical Causes Scale were utilized to assess the awareness of CC symptoms, risk factors, and causation myths. A total of 7058 participants were included. Only 27.4%, 23.6%, and 8.1% exhibited high awareness of CC symptoms, risk factors, and causation myths, respectively. Women with high awareness of CC causation myths were more likely to exhibit positive attitudes toward CC than those with low awareness (OR = 1.83, 95% CI 1.51–2.23). However, there were no associations between displaying high awareness of CC symptoms and risk factors and showing positive attitudes toward CC. Good understanding of CC causation seems to be positively associated with exhibiting favorable attitudes toward the disease. Educational initiatives may prioritize enhancing public awareness of CC causation to optimize the cultivation of positive attitudes.

**Supplementary Information:**

The online version contains supplementary material available at 10.1038/s41598-025-08068-1.

## Introduction

Cervical cancer (CC) is the fourth most common cancer among women worldwide, accounting for 661,021 estimated new cases and 348,189 deaths in 2022 per GLOBOCAN 2022^1–4^. CC mortality exhibits varying distribution patterns worldwide, with over 85.0% of fatalities occurring in low- and middle-income countries^5^. In contrast, there has been a remarkable and consistent decline in CC incidence and mortality rates over the last five decades in high-income countries^6^. This positive shift can be attributed to efficient preventive measures such as prophylactic human papillomavirus vaccination, screening, and early treatment of precancerous lesions^7–9^.

In Palestine, CC is the third most common gynecological cancer^10^. It has an age-standardized mortality rate of 1.9 per 100,000 females^11^. This rate is slightly higher than that of other Middle Eastern countries, such as Jordan (1.8 per 100,000 females) and Saudi Arabia (1.5 per 100,000 females)^11^. Nonetheless, a concerning trend is evident in Arab countries, where women often present with CC at an advanced stage^12^. This may be attributed to factors such as poor socioeconomics, low awareness levels, and fragile healthcare systems^13^. Recognizing and addressing these factors is essential for effectively developing strategies for the prevention and early detection of CC.

Cervical cancer is a preventable disease by the combination of vaccination of 9–14 year old girls and boys against human papillomavirus, national screening programs from 25–30 years and older to detect precancerous changes and by early treatment of disease if these measures have failed^6,7,9^. In fact, a 90% reduction of HPV-related CC causation has already been achieved globally by HPV vaccination programs^14^. However, currently, in Palestine, neither HPV vaccination programs nor CC screening programs exist. This increases the importance of CC awareness and attitudes towards CC.

The term “attitude” refers to a person’s response and approach to their health condition. It includes a person’s cognitive, emotional, and behavioral responses to their illness^15^. Notably, attitudes may influence how people perceive and interpret their symptoms, treatment options, and the impact of illness on their lives^16^. Awareness of cancer has emerged as a critical determinant that can influence people’s attitudes toward the disease and has a significant impact on their likelihood of taking appropriate and timely healthcare actions^17^. Nevertheless, previous studies from Palestine have shown poor awareness of CC^18–20^. However, there is a lack of studies evaluating the interplay of awareness regarding CC symptoms, risk factors, and causation myths and attitudes toward the disease among Palestinian women. It is critical to fill this gap in the literature because attitudes toward illness significantly influence health behaviors, including the likelihood of participating in preventive measures and seeking early treatment^21,22^.

Therefore, this study aimed to address the knowledge gap with regards to examining the relationship between Palestinian women’s awareness of CC signs/symptoms, risk factors, and causation myths, and their attitudes toward the disease. In particular, the research question was whether awareness level of different aspects of CC (signs/symptoms, risk factors, and causation myths) can be associated with attitudes toward CC. By answering this question, this study can provide valuable insights into how awareness campaigns can be more effectively tailored to not only promote awareness but also positively influence attitudes, thereby potentially improving preventive behaviors and early diagnoses among Palestinian women.

## Methods

### Study design, setting, and population

This was a nationwide cross-sectional study conducted between July 2019 and March 2020 in Palestine. Palestinian women over the age of 18, representing more than half of the female population in Palestine^23^, were targeted in this study. In our study, 62.4% of the participating females were from the West Bank and Jerusalem, and 37.6% from Gaza, which can be compared to the real-world distribution of females in Palestine: women aged 20 and above constitute more than 50.2% of the female population in the West Bank and 48.9% in Gaza^24^. Of the 16 Palestinian governorates, 11 governorates—seven in the West Bank and Jerusalem and four in the Gaza Strip—were used to recruit adult women. Adult Palestinian women visiting the designated data collection sites were considered eligible for participation. Women of non-Palestinian nationality, those employed or studying in health-related fields, those who attended oncology departments during data collection, and those unable to complete the questionnaire were all excluded from the study.

### Sampling methods

Convenience sampling was utilized to enroll eligible women from a diverse range of settings, including governmental hospitals, primary healthcare facilities, and public spaces such as shopping centers, markets, parks, restaurants, mosques, churches, and transportation stations. Those data collection sites were strategically spread across different locations in Palestine to ensure a broad representation of the Palestinian community in the study cohort^18–20^. Women were approached in the data collection sites by trained female data collectors from a medical background. The study and its purpose were explained to them, and it was checked if they would be eligible to participate. If so, they were asked if they were willing to complete the questionnaire.

### Study population

**Fig. 1:**
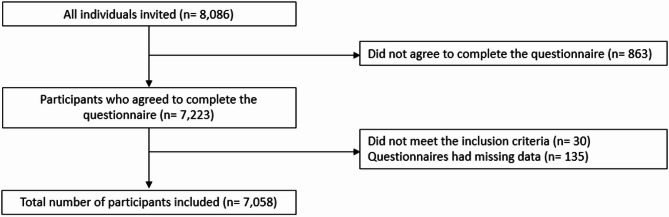
Flowchart for selecting the study cohort.

In total, 8086 individuals were approached, of whom a total of 7223 participants completed the questionnaire, resulting in a response rate of 89.3%. The final analysis included 7058 questionnaires; 30 questionnaires were excluded because they did not meet the inclusion criteria, and 135 had missing values (Fig. 1).

### Questionnaire and data collection

A modified, translated-into-Arabic version of the CC Awareness Measure (CeCAM), which is a validated questionnaire developed to measure awareness of CC in the general population, was used for data collection^25^. In addition, a modified, translated-into-Arabic version of the Cancer Awareness Measure-Mythical Causes Scale (CAM-MYCS) was used to assess the awareness of myths about CC causation^26^. The questionnaires were translated from English to Arabic by two healthcare professionals fluent in both languages. After that, the Arabic version was back-translated to English by another two bilingual healthcare professionals. These healthcare professionals had relevant medical and research experience in gynecologic oncology, public health, and survey design. The questionnaire’s content validity was evaluated by five independent healthcare professionals and researchers. A pilot study involving 130 participants was carried out to assess the clarity of the questions in the Arabic version of the questionnaire. The responses gathered in the pilot study were excluded from the final analysis. The questionnaire demonstrated acceptable internal consistency, with a Cronbach’s alpha of 0.75, as compared to the original CeCAM (α = 0.85).

The study questionnaire is provided in Supplementary file 1. It consisted of five sections. The first section included nine questions about the sociodemographic characteristics of the study participants, including age, marital status, level of education, occupation, monthly income, place of residency, history of a chronic disease, knowledge of someone with cancer, and site of data collection. The second section evaluated the participants’ ability to recognize 12 CC symptoms based on a 5-point Likert scale (1 = strongly disagree, 5 = strongly agree). Of those 12 symptoms, 11 were adapted from the original CeCAM^25^, and ‘extreme generalized fatigue’ was added, as it was included in other forms of the Cancer Awareness Measure^27,28^, and including it in the context of CC was thought to be helpful. The 5-point Likert scale items substituted the yes/no/unknown questions in the original CeCAM to reduce the likelihood of participants responding at random. Consequently, the responses from the participants were transformed into correct and incorrect responses, as described by prior studies^18–20^.

Consistent with the original CeCAM^25^, the third section comprised 11 questions based on a 5-point Likert scale (1 = strongly disagree, 5 = strongly agree) to assess the knowledge of CC risk factors. A few items were modified in the Arabic version to make them more culturally accepted in Palestine. In particular, ‘having a sexual partner with many previous partners’ was modified to ‘having a husband with many previous partners’. Similarly, ‘starting to have sex at a young age (before age 17)’ was modified to ‘being married at a young age (before age 17)’. In addition, ‘having a sexual partner who is not circumcised’ was modified to ‘having a husband who is not circumcised’. The fourth section assessed the recognition of 13 myths about CC causation as incorrect. All but one of the examined myths were adopted from the original CAM-MYCS^26^. The item ‘eating burnt food’ was deemed important and was added. The fifth section included 11 questions related to attitudes toward CC that were adapted from previous studies^29–32^ and utilized the same aforementioned 5-point Likert scale.

Face-to-face interviews with eligible participants were carried out by well-trained female data collectors with medical backgrounds. The selection of female data collectors was intended to facilitate the answering of some possibly sensitive questions. The data collection was conducted using the secure and easy-to-use tool Kobo Toolbox, which is accessible via smartphones^33^.

### Statistical analysis

Participant characteristics were summarized using descriptive statistics. Non-normally distributed continuous variables were described using the median and interquartile range (IQR), while categorical variables were summarized using frequencies and percentages. Based on the minimum wage in Palestine (1450 NIS, approximately $390)^34^, monthly income was dichotomized as < 1450 NIS and ≥ 1450 NIS.

Recognition of CC symptoms and risk factors was evaluated using a 5-point Likert scale (1 = strongly disagree, 5 = strongly agree). Responses of ‘strongly agree’ or ‘agree’ were considered correct, while ‘strongly disagree’, ‘disagree’, and ‘not sure’ were considered incorrect. Additionally, participants were assessed on their awareness of CC causation myths, and answers expressing disagreement (‘disagree’ or ‘strongly disagree’) were considered correct, whereas all other responses were considered incorrect.

In consistence with previous studies^18,20,21^, a scoring system was utilized to assess the awareness level of CC symptoms, risk factors, and causation myths. Participants were given one point for each correctly identified item. Subsequently, the cumulative awareness score for each domain was computed and stratified into tertiles. The top tertile denoted ‘high’ awareness, while the remaining two tertiles were designated ‘low’ awareness. Similarly, participants were given one point for responding with “agree” or “strongly agree” on each of the questions related to attitudes toward CC. The total attitude score was calculated, and the median was utilized to dichotomize it; a score ≤ 4 was considered a ‘negative’ attitude, and a score ≥ 5 was considered a ‘positive’ attitude.

The associations between demonstrating high awareness in each domain (CC symptoms, risk factors, and causation myths) and agreement with questions related to positive attitudes toward CC were examined utilizing Pearson’s Chi-square test. Multivariable logistic regression analysis was subsequently conducted to adjust for several covariates, including age, educational level, employment status, monthly income, marital status, place of residency, presence of a chronic disease, familiarity with someone diagnosed with cancer, and site of data collection. The selection of these covariates was predetermined based on prior studies^18,20,21^. Similar analyses were conducted to investigate the association between demonstrating high awareness in each domain and exhibiting positive attitudes toward CC.

Missing data were hypothesized to be missed completely at random. Therefore, complete case analysis was utilized. The data were analyzed using Stata software version 17.0 (StataCorp, College Station, Texas, United States).

## Results

### Participant characteristics

The median age [IQR] of all participants was 32.0 [24.0, 42.0] years, ranging from 18 to 87 years. More than half of the study participants (n = 3893, 55.2%) had only completed secondary education or below, and around two-thirds of the participants (n = 4666, 66.1%) had low monthly income (Table 1).

**Table 1:** Characteristics of study participants.

Characteristic	Total (n = 7058)
Age, median [IQR]	32.0 [24.0, 42.0]
Educational level, n (%)	
Secondary or below	3893 (55.2)
Above secondary	3165 (44.8)
Occupation, n (%)	
Housewife	4647 (65.8)
Employed	1476 (20.9)
Retired	69 (1.0)
Student	866 (12.3)
Monthly income ≥ 1450 NIS, n (%)	4666 (66.1)
Marital status, n (%)	
Single	1657 (23.5)
Married	5058 (71.6)
Divorced/Widowed	343 (4.9)
Residency, n (%)	
Gaza Strip	2655 (37.6)
West Bank and Jerusalem	4403 (62.4)
Having a chronic disease, n (%)	1397 (19.8)
Knowing someone with cancer, n (%)	4083 (57.9)
Site of data collection, n (%)	
Public spaces	2.695 (38.2)
Hospitals	1890 (26.7)
Primary healthcare centers	2473 (35.1)

n = number of participants, IQR = interquartile range.

### CC symptom awareness and attitudes toward CC.

A total of 1934 participants (27.4%) demonstrated high awareness of CC symptoms (Table 2). Participants with a high awareness of CC symptoms were significantly more likely to agree on four out of 11 questions, namely, ‘early detection of CC increases the possibility of more effective treatment’ (OR = 2.56, 95% CI: 2.02- 3.25), ‘early detection of CC increases the chances of survival’ (OR = 2.46, 95% CI: 2.01- 3.02), ‘CC is not an infectious disease’ (OR = 1.27, 95% CI: 1.12- 1.44), and ‘if you developed CC, you would live longer than 5 years’ (OR = 1.23, 95% CI: 1.15- 1.45).

**Table 2:** Summary of the association between demonstrating high awareness of cervical cancer symptoms and showing positive attitudes toward cervical cancer among study participants.

Question	Low awareness (N = 5124) n (%)	High awareness (N = 1934) n (%)	OR* (95% CI)	p–value
Early detection of cervical cancer increases the possibility of more effective treatment	4533 (88.5)	1850 (95.7)	2.56 (2.02- 3.25)	** < 0.001**
Early detection of cervical cancer increases the chances of survival	4361 (85.1)	1814 (93.8)	2.46 (2.01- 3.02)	** < 0.001**
Cervical cancer is not an infectious disease	3660 (71.4)	1501 (77.6)	1.27 (1.12- 1.44)	** < 0.001**
Taking herbs is not a cure for cervical cancer	2173 (42.4)	835 (43.2)	1.03 (0.92- 1.14)	0.65
Cervical cancer would not threaten your relationship with your (future) spouse	1738 (33.9)	608 (31.4)	0.87 (0.77- 0.97)	**0.015**
The problems that you would experience with cervical cancer would not last for a long time	2112 (41.2)	726 (37.5)	0.84 (0.75- 0.93)	** < 0.001**
Your chances of getting cervical cancer in the next few years are not high	1938 (37.5)	689 (35.6)	0.94 (0.84- 1.05)	0.24
The thought of cervical cancer does not scare you	1387 (27.1)	532 (27.5)	1.02 (0.91- 1.15)	0.70
If you developed cervical cancer, you would not feel that the therapy makes you sicker than the disease itself	1361 (26.6)	499 (25.8)	0.95 (0.84- 1.07)	0.43
You will not get cervical cancer sometime during your life	1640 (32.0)	571 (29.5)	0.92 (0.82- 1.03)	0.14
If you developed cervical cancer, you would live longer than 5 years	1407 (27.5)	620 (32.1)	1.23 (1.15- 1.45)	** < 0.001**

n = number of participants, OR = odds ratio, CI = confidence interval.

*Adjusted for age, educational level, employment status, monthly income, marital status, place of residency, presence of a chronic disease, familiarity with someone diagnosed with cancer, and site of data collection.

p-values in bold are less than 0.05.

The most observed agreement to questions related to attitudes toward CC among participants with high (n = 1850, 95.7%) or low (n = 4533, 88.5%) CC symptom awareness was for the belief that ‘early detection of CC increases the possibility of more effective treatment’ followed by ‘early detection of CC increases the chances of survival’ (high awareness group: n = 1814, 93.8%; low awareness group: n = 4361, 85.1%). In contrast, the least commonly observed agreement among participants with high (n = 499, 25.8%) and low (n = 1361, 26.6%) awareness were for ‘if you developed CC, you would not feel that the therapy makes you sicker than the disease itself’.

### CC risk factor awareness and attitudes toward CC

A total of 1670 participants (23.6%) exhibited high awareness of CC risk factors (Table 3). Participants with high awareness of CC risk factors were significantly more likely to regard three specific questions out of the 11 questions related to attitudes toward CC, as correct. These three questions were ‘early detection of CC increases the possibility of more effective treatment’ (OR = 1.63, 95% CI: 1.31- 2.03), ‘early detection of CC increases the chances of survival’ (OR = 1.60, 95% CI: 1.32- 1.94), and ‘if you developed CC, you would live longer than 5 years’ (OR = 1.37, 95% CI: 1.22- 1.55).

**Table 3:** Summary of association between demonstrating high awareness of cervical cancer risk factors and showing positive attitudes toward cervical cancer among study participants.

Question	Low awareness (N = 5388) n (%)	High awareness (N = 1670) n (%)	OR* (95% CI)	p–value
Early detection of cervical cancer increases the possibility of more effective treatment	4819 (89.4)	1564 (93.7)	1.63 (1.31- 2.03)	** < 0.001**
Early detection of cervical cancer increases the chances of survival	4649 (86.3)	1526 (91.4)	1.60 (1.32- 1.94)	** < 0.001**
Cervical cancer is not an infectious disease	3977 (73.8)	1184 (70.9)	0.82 (0.72- 0.93)	** < 0.001**
Taking herbs is not a cure for cervical cancer	2359 (43.8)	649 (38.9)	0.81 (0.72- 0.91)	** < 0.001**
Cervical cancer would not threaten your relationship with your (future) spouse	1836 (34.1)	510 (30.5)	0.86 (0.76- 0.97)	**0.014**
The problems that you would experience with cervical cancer would not last for a long time	2237 (41.5)	601 (36.0)	0.78 (0.69- 0.87)	** < 0.001**
Your chances of getting cervical cancer in the next few years are not high	2033 (37.7)	594 (35.6)	0.92 (0.82- 1.03)	0.17
The thought of cervical cancer does not scare you	1472 (27.3)	447 (26.8)	0.98 (0.86- 1.10)	0.69
If you developed cervical cancer, you would not feel that the therapy makes you sicker than the disease itself	1426 (26.5)	434 (26.0)	0.98 (0.86- 1.11)	0.74
You will not get cervical cancer sometime during your life	1693 (31.4)	518 (31.6)	1.00 (0.89- 1.13)	0.99
If you developed cervical cancer, you would live longer than 5 years	1466 (27.2)	561 (33.6)	1.37 (1.22- 1.55)	**0.001**

n = number of participants, OR = odds ratio, CI = confidence interval.

*Adjusted for age, educational level, employment status, monthly income, marital status, place of residency, presence of a chronic disease, familiarity with someone diagnosed with cancer, and site of data collection.

p-values in bold are less than 0.05.

The highest level of agreement among participants, whether they demonstrated high (n = 1564, 93.7%) or low (n = 4819, 89.4%) awareness of CC risk factors, was found for the belief that ‘early detection of CC increases the possibility of more effective treatment,’ followed by ‘early detection of CC increases the chances of survival’ (high awareness group: n = 1526, 91.4%; low awareness group: n = 4649, 86.3%). Conversely, the least commonly observed agreement among both groups, whether high (n = 434, 26.0%) or low (n = 1426, 26.5%) awareness, was for ‘if you developed CC, you would not feel that the therapy makes you sicker than the disease itself’.

### CC causation myth awareness and attitudes toward CC

Only 575 participants (8.1%) exhibited high awareness of myths surrounding CC causation (Table 4). Participants with high awareness of CC causation myths were more likely to agree on 8 out of 11 questions regarding attitudes toward CC, namely ‘CC is not an infectious disease’ (OR = 1.31, 95% CI: 1.07- 1.60), ‘taking herbs is not a cure for CC’ (OR = 1.71, 95% CI: 1.44- 2.05), ‘CC would not threaten your relationship with your (future) spouse’ (OR = 1.36, 95% CI: 1.13- 1.63), ‘the problems that you would experience with CC would not last for a long time’ (OR = 1.47, 95% CI: 1.23- 1.75), ‘the thought of CC does not scare you’ (OR = 1.37, 95% CI: 1.14- 1.65), ‘if you developed CC, you would not feel that the therapy makes you sicker than the disease itself’ (OR = 1.55, 95% CI: 1.29- 1.87), and ‘you will not get CC during your life’ (OR = 1.50, 95% CI: 1.26- 1.80).

**Table 4:** Summary of association between demonstrating high awareness of cervical cancer causation myths and showing positive attitudes toward cervical cancer among study participants.

Question	Low awareness (N = 6483) n (%)	High awareness (N = 575) n (%)	OR* (95% CI)	p–value
Early detection of cervical cancer increases the possibility of more effective treatment	5883 (90.7)	500 (87.0)	0.72 (0.55- 0.94)	**0.017**
Early detection of cervical cancer increases the chances of survival	5697 (87.9)	478 (83.1)	0.72 (0.57- 0.91)	**0.006**
Cervical cancer is not an infectious disease	4727 (72.9)	434 (75.5)	1.31 (1.07- 1.60)	**0.010**
Taking herbs is not a cure for cervical cancer	2683 (41.4)	325 (56.5)	1.71 (1.44- 2.05)	** < 0.001**
Cervical cancer would not threaten your relationship with your (future) spouse	2144 (33.1)	202 (35.1)	1.36 (1.13- 1.63)	**0.001**
The problems that you would experience with cervical cancer would not last for a long time	2568 (39.6)	270 (47.0)	1.47 (1.23- 1.75)	** < 0.001**
Your chances of getting cervical cancer in the next few years are not high	2327 (35.9)	300 (52.2)	1.73 (1.45- 2.06)	** < 0.001**
The thought of cervical cancer does not scare you	1731 (26.7)	188 (32.7)	1.37 (1.14- 1.65)	**0.001**
If you developed cervical cancer, you would not feel that the therapy makes you sicker than the disease itself	1669 (25.7)	191 (33.2)	1.55 (1.29- 1.87)	** < 0.001**
You will not get cervical cancer sometime during your life	1965 (30.3)	246 (42.8)	1.50 (1.26- 1.80)	** < 0.001**
If you developed cervical cancer, you would live longer than 5 years	1866 (28.8)	161 (28.0)	0.89 (0.73- 1.08)	0.22

n = number of participants, OR = odds ratio, CI = confidence interval.

*Adjusted for age, educational level, employment status, monthly income, marital status, place of residency, presence of a chronic disease, familiarity with someone diagnosed with cancer, and site of data collection.

p-values in bold are less than 0.05.

Regardless of their awareness of CC causation, most participants agreed that ‘early detection of CC increases the possibility of more effective treatment’ (high awareness group: n = 500, 87.0%; low awareness group: n = 5883, 90.7%). In contrast, the least frequent agreement was for 'if you developed CC, you would live longer than 5 years’ among participants with high awareness (n = 161, 28%), whereas it was for 'if you developed CC, you would not feel that the therapy makes you sicker than the disease itself’ among those with low awareness (n = 1669, 25.7%).

### Association between high CC awareness and positive attitudes toward CC

Participants with high awareness of CC causation myths were more likely to exhibit positive attitudes toward CC than were those with low awareness (OR = 1.83, 95% CI 1.51- 2.23; Table 5). However, no independent associations were found between exhibiting high awareness of each of CC symptoms or risk factors and showing positive attitudes toward the disease.

**Table 5:** Association of demonstrating high awareness in each domain with showing positive attitudes toward cervical cancer.

Attitudes toward CC	Low awareness of CC symptoms (N = 5124) n (%)	High awareness of CC symptoms (N = 1934) n (%)	OR* (95% CI)	p–value
Negative Positive	1935 (37.8) 3189 (62.2)	675 (34.9) 1259 (65.1)	1.10 (0.98-1.22)	0.11

Attitudes toward CC	Low awareness of CC risk factors (N = 5388) n (%)	High awareness of CC risk factors (N = 1670) n (%)	OR (95% CI)	p–value
Negative Positive	1980 (36.7) 3408 (63.3)	630 (37.7) 1040 (62.3)	0.94 (0.84-1.06)	0.33

Attitudes toward CC	Low awareness of CC causation myths (N = 6483) n (%)	High awareness of CC causation myths (N = 575) n (%)	OR (95% CI)	p–value
Negative Positive	2461 (38.0) 4022 (62.0)	149 (25.9) 426 (74.1)	1.83 (1.51-2.23)	** < 0.001**

n = number of participants, OR = odds ratio, CI = confidence interval, CC = cervical cancer.

*Adjusted for age, educational level, employment status, monthly income, marital status, place of residency, presence of a chronic disease, familiarity with someone diagnosed with cancer, and site of data collection.

p-values in bold are less than 0.05.

## Discussion

Overall, this study showed that the majority of women had a low level of awareness regarding CC, with only 27.4%, 23.6%, and 8.1% of women exhibiting good awareness of symptoms, risk factors, and causation myths, respectively. Similarly, a hospital-based study in India also highlighted poor knowledge regarding CC, with 64% of participants being unaware of any early symptoms and only 39% aware of at least one risk factor. Despite this limited knowledge, the Indian study reported a notably positive attitude among women, with 76.2% expressing willingness to undergo screening if it were offered free of cost, even though only 9.5% had ever been screened^32^. In this current study, most participants, regardless of their awareness level, acknowledged the importance of early detection in improving survival rates and the effectiveness of treatment. This further underscores the need for a national screening program and the inclusion of HPV vaccination in immunization programs as essential preventive strategies. This is especially important given the insidious nature of CC, which often remains asymptomatic in its initial stages, and most cases present at advanced stages^35^. In line with this, Brisson and colleagues provided evidence from their international study that a 90% HPV vaccination coverage of girls can lead to CC elimination in most low-income and lower-middle-income countries within the next century. The authors also highlighted the vital role of screening programs in accelerating the elimination process by 11–31 years^36^. In our study, women with a higher awareness of CC causation myths were also more likely to exhibit a positive attitude toward the disease, reinforcing the observation that positive attitudes can exist even in the presence of low awareness levels. However, no association was found between having a high awareness of CC symptoms or risk factors and displaying positive attitudes toward the disease.

Understanding the interplay between CC awareness and attitudes is crucial because it may impact healthcare decisions and outcomes. People’s actions when seeking healthcare could be influenced by their knowledge and views regarding the presentation and etiology of a given illness^37^. In the context of CC, a positive attitude toward the disease may motivate women to adopt healthy habits, such as taking the vaccine and undergoing screening. Moreover, patients who maintain a positive attitude during illness or recovery tend to have better outcomes than those who display negative attitudes^38^. Furthermore, a study in Saudi Arabia reported a median awareness score of 40%, a figure comparable to or lower than those from other Gulf countries such as Oman (38.3%), Kuwait (54%), and Sharjah (66.2%)^39–42^. While CC awareness levels in our study (27.4% for symptoms and 23.6% for risk factors) are somewhat lower than those in the Gulf region, they fall within a similar range, highlighting a regional trend of insufficient awareness. These findings emphasize the urgent need for targeted education and screening initiatives across the region, including Palestine.

In this study, a minority of study participants agreed that they would not feel that the therapy made them sicker than developing CC itself. A possible contributing factor to this could be personal experiences or stories heard from others about CC treatment, especially in low-resource settings, such as Palestine^20^. Furthermore, there could be limited exposure to accurate information about the diagnosis and treatment outcomes of CC^43^. Improving clinician-patient communication and creating targeted interventions that address these concerns and misconceptions is critical, ultimately fostering a more positive attitude toward CC treatment^44^.

This study revealed that having a high awareness level of myths about CC causation was associated with positive attitudes toward the disease. A possible explanation for this could be that those with a high awareness of CC causation may seek additional information to understand the true causes, and they are more likely to take a proactive approach to early detection and prevention, as they realize that anyone, regardless of lifestyle or background, can develop CC^45^.

On the other hand, there was no independent association between high awareness of CC symptoms or risk factors and attitudes toward CC. Attitudes toward a disease are multifaceted and usually shaped by various factors beyond knowledge of symptoms and risk factors, such as personal experiences and cultural beliefs^46^. In addition, emotions such as optimism, empowerment, and support can contribute to positive attitudes toward cancer^47^. However, symptom awareness is mostly based on clinical knowledge and manifestations of the disease. Therefore, having a positive attitude may motivate women to seek medical attention when symptoms emerge, but it does not guarantee a thorough comprehension of the symptoms.

### Future directions

Based on the findings of this study, there is a need to implement comprehensive education programs that cover all aspects of CC, including symptoms, risk factors, causation myths, and prevention methods (such as vaccination and screening).To ensure widespread dissemination, these initiatives should be integrated into routine visits to primary care doctors, gynecology clinics, high schools, and online platforms.

Community engagement may play a role in improving the relationship between CC awareness and attitudes. This could be achieved by involving community leaders, advocacy groups, and peer support groups in spreading knowledge about CC and offering help and support to affected women. Furthermore, it is imperative to implement immediate policy initiatives to enhance the prevention, early detection, and treatment of CC. This includes the establishment of policies that allocate funds for research, screening programs, and immunization campaigns.

### Limitations

The utilization of convenience sampling may not adequately ensure the creation of a representative sample of the Palestinian population, which may limit the generalizability of the results. Nonetheless, the data collection from diverse locations, the large sample size, and the high response rate may have mitigated this. Additionally, the decision to exclude individuals from oncology departments and those with medical backgrounds might have resulted in a diminished number of participants presumed to possess high CC awareness. However, this exclusion was intended to enhance the study’s measurement of public awareness of CC. At the time of the study, the CeCAM and CAM-MYCS were not available in Arabic and had also not been formally validated in an Arabic version. The lack of use of previously validated tools may cause some biases. However, content validation, a careful back-to-back translation, and internal consistency measurement were performed prior to the use of the instrument by a team of experts in the fields as well as fluent speakers of both languages. In the attempt to produce culturally sensitive data collection tools for the data collection in Palestine, the wording of some questions was changed slightly, such as replacement of the word ‘partner’ with ‘husband’ or ‘spouse’. This was done with the intention of producing a more inclusive tool in the context of Palestine. This might have led to selection bias or difficulties to complete the questionnaires to other women. Furthermore, it is important to acknowledge that the study focused on participants’ perceived knowledge and did not evaluate the awareness of individuals displaying actual CC symptoms.

### Conclusion

This study revealed significant gaps in Palestinian women’s knowledge concerning CC symptoms, risk factors, and causation myths. Women who were more aware of CC causation myths had a greater likelihood of exhibiting positive attitudes toward the disease. This indicates the need for effective measures to disseminate proper information that facilitates the shaping of positive attitudes toward CC among Palestinian women and sets the stage for comprehensive national HPV vaccination and CC screening programs in Palestine.

## Electronic supplementary material

Below is the link to the electronic supplementary material.

Supplementary Material 1

## Data Availability

The dataset used and analyzed during the current study is available by the corresponding author upon reasonable request.

## Abbreviations

CC: Cervical cancer
CeCAM: CC Awareness Measure
CAM-MYCS: Cancer Awareness Measure-Mythical Causes Scale
CI: Confidence interval
OR: Odds ratio
IQR: Interquartile range
LMICs: low-income and lower-middle-income countries
