# Supplementary material for: Assessing the role of cervical cancer awareness in shaping attitudes toward the disease among Palestinian women
Source: Sci Rep. 2025 Jul 1;15:21530. doi: 10.1038/s41598-025-08068-1 (PMC12214724; doi:10.1038/s41598-025-08068-1)
Supplement: Supplementary file 1 — Supplementary Material 1 [file 41598_2025_8068_MOESM1_ESM.docx]

**Cervical Cancer Awareness and Attitudes Questionnaire**

Serial number: ………. Location: …………. Governorate: ……….

1. **Sociodemographic Data**

- Age: …….. years
- Marital status: 🞏 Single 🞏 Married 🞏 Divorced 🞏 Widowed
- Highest level of education: 🞏 Secondary 🞏 Post-secondary
- Occupation: 🞏 Housewife/unemployed 🞏 Employed 🞏 Retired 🞏 Student
- Monthly income (NIS): ………………
- Do you have any chronic disease? 🞏 No 🞏 Yes
- Have any of your family or close friends had cancer? 🞏 No 🞏 Yes

**2. Awareness of Cervical Cancer Symptoms**

| Table (1): The following may or may not be warning signs for cervical cancer. We are interested in your opinion: | | | | | |
| --- | --- | --- | --- | --- | --- |
| Symptom | **1= Strongly disagree** | **2= Disagree** | **3= Not sure** | **4= Agree** | **5= Strongly agree** |
| 1. Vaginal bleeding between periods |  |  |  |  |  |
| 1. Persistent lower back pain |  |  |  |  |  |
| 1. Persistent vaginal discharge that smells unpleasant |  |  |  |  |  |
| 1. Discomfort or pain during sex |  |  |  |  |  |
| 1. Having menstrual periods that are heavier or longer than usual |  |  |  |  |  |
| 1. Persistent diarrhea |  |  |  |  |  |
| 1. Vaginal bleeding after the menopause |  |  |  |  |  |
| 1. Persistent pelvic pain |  |  |  |  |  |
| 1. Vaginal bleeding during or after sex |  |  |  |  |  |
| 1. Blood in the stool or urine |  |  |  |  |  |
| 1. Unexplained weight loss |  |  |  |  |  |
| 1. Extreme fatigue |  |  |  |  |  |

**3. Awareness of Cervical Cancer Risk Factors**

| Table (2): How much do you agree that each of these can increase a woman’s chance of developing cervical cancer? | | | | | |
| --- | --- | --- | --- | --- | --- |
| Factor | **1= Strongly disagree** | **2= Disagree** | **3= Not sure** | **4= Agree** | **5= Strongly agree** |
| 1. Infection with human papillomavirus (HPV) |  |  |  |  |  |
| 1. Smoking any cigarettes at all |  |  |  |  |  |
| 1. Having a weakened immune system (e.g. immunosuppressant drugs or having a transplant) |  |  |  |  |  |
| 1. Long term use of the contraceptive pill |  |  |  |  |  |
| 1. Having many children |  |  |  |  |  |
| 1. Infection with a sexually transmitted infection (e.g, Chlamydia) |  |  |  |  |  |
| 1. Being married at young age (before age 17) |  |  |  |  |  |
| 1. Having a husband who is not circumcised |  |  |  |  |  |
| 1. Having a sexual partner with many previous partners |  |  |  |  |  |
| 1. Having a relative with cervical cancer |  |  |  |  |  |
| 1. Not going for regular smear (Pap) tests |  |  |  |  |  |

**D. Awareness of Causation Myths**

| Table (3): The following practices may or may not cause cervical cancer. We are interested in your opinion. | | | | | |
| --- | --- | --- | --- | --- | --- |
| Practice | **1= Strongly Disagree** | **2= Disagree** | **3= Not Sure** | **4= Agree** | **5= Strongly agree** |
| 1. Drinking from plastic bottles |  |  |  |  |  |
| 1. Eating food containing artificial sweeteners |  |  |  |  |  |
| 1. Eating genetically modified food |  |  |  |  |  |
| 1. Eating food containing additives |  |  |  |  |  |
| 1. Using microwave ovens |  |  |  |  |  |
| 1. Using aerosol containers |  |  |  |  |  |
| 1. Using mobile phones |  |  |  |  |  |
| 1. Using cleaning products |  |  |  |  |  |
| 1. Living near power lines |  |  |  |  |  |
| 1. Feeling stressed |  |  |  |  |  |
| 1. Having a physical trauma |  |  |  |  |  |
| 1. Exposure to electromagnetic frequencies (e.g, Wi-Fi and Radio/TV frequencies) |  |  |  |  |  |
| 1. Eating burnt food (e.g., bread or barbeque) |  |  |  |  |  |

**5. Attitudes toward Cervical Cancer**

| Table (4): We are interested in your opinion on the following questions: | | | | | |
| --- | --- | --- | --- | --- | --- |
| Question | **1= Strongly disagree** | **2= Disagree** | **3= Not sure** | **4= Agree** | **5= Strongly agree** |
| 1. Early detection of cervical cancer increases the possibility of more effective treatment. |  |  |  |  |  |
| 1. Early detection of cervical cancer increases the chances of survival. |  |  |  |  |  |
| 1. Cervical cancer is not an infectious disease. |  |  |  |  |  |
| 1. Taking herbs is not a cure for cervical cancer. |  |  |  |  |  |
| 1. Cervical cancer would not threaten your relationship with your (future) spouse. |  |  |  |  |  |
| 1. The problems that you would experience with cervical cancer would not last for a long time. |  |  |  |  |  |
| 1. Your chances of getting cervical cancer in the next few years are not high. |  |  |  |  |  |
| 1. The thought of cervical cancer does not scare you. |  |  |  |  |  |
| 1. If you developed cervical cancer, you would not feel that the therapy makes you sicker than the disease itself. |  |  |  |  |  |
| 1. You will not get cervical cancer sometime during your life. |  |  |  |  |  |
| 1. If you developed cervical cancer, you would live longer than 5 years. |  |  |  |  |  |
